# Large-scale bioreactor production of extracellular vesicles from mesenchymal stromal cells for treatment of acute radiation syndrome

**DOI:** 10.1186/s13287-024-03688-2

**Published:** 2024-03-13

**Authors:** John A. Kink, Michael A. Bellio, Matthew H. Forsberg, Alexandra Lobo, Anna S. Thickens, Bryson M. Lewis, Irene M. Ong, Aisha Khan, Christian M. Capitini, Peiman Hematti

**Affiliations:** 1 Department of Medicine, School of Medicine and Public Health, University of Wisconsin, Madison, WI USA; 2 University of Wisconsin Carbone Cancer Center, 1111 Highland Ave, WIMR 4137, Madison, WI USA; 3 Interdisciplinary Stem Cell Institute, University of Miami, Miller School of Medicine, Miami, FL USA; 4 Department of Pediatrics, School of Medicine and Public Health, University of Wisconsin, Madison, WI USA; 5 Department of Biostatistics and Medical Informatics, School of Medicine and Public Health, University of Wisconsin, Madison, WI USA; 6 Department of Obstetrics and Gynecology, School of Medicine and Public Health, University of Wisconsin, Madison, WI USA; 7 Medical College of Wisconsin, 9200 W. Wisconsin Ave, Milwaukee, WI 53326 USA

**Keywords:** Extracellular vesicles, Exosomes, Mesenchymal stromal cells, TLR4, Acute radiation syndrome

## Abstract

**Background:**

Hematopoietic acute radiation syndrome (H-ARS) occurring after exposure to ionizing radiation damages bone marrow causing cytopenias, increasing susceptibility to infections and death. We and others have shown that cellular therapies like human mesenchymal stromal cells (MSCs), or monocytes/macrophages educated ex-vivo with extracellular vesicles (EVs) from MSCs were effective in a lethal H-ARS mouse model. However, given the complexity of generating cellular therapies and the potential risks of using allogeneic products, development of an “off-the-shelf” cell-free alternative like EVs may have utility in conditions like H-ARS that require rapid deployment of available therapeutics. The purpose of this study was to determine the feasibility of producing MSC-derived EVs at large scale using a bioreactor and assess critical quality control attributes like identity, sterility, and potency in educating monocytes and promoting survival in a lethal H-ARS mouse model.

**Methods:**

EVs were isolated by ultracentrifugation from unprimed and lipopolysaccharide (LPS)-primed MSCs grown at large scale using a hollow fiber bioreactor and compared to a small scale system using flasks. The physical identity of EVs included a time course assessment of particle diameter, yield, protein content and surface marker profile by flow-cytometry. Comparison of the RNA cargo in EVs was determined by RNA-seq. Capacity of EVs to generate exosome educated monocytes (EEMos) was determined by qPCR and flow cytometry, and potency was assessed in vivo using a lethal ARS model with NSG mice.

**Results:**

Physical identity of EVs at both scales were similar but yields by volume were up to 38-fold more using a large-scale bioreactor system. RNA-seq indicated that flask EVs showed upregulated let-7 family and miR-143 micro-RNAs. EEMos educated with LPS-EVs at each scale were similar, showing increased gene expression of IL-6, IDO, FGF-2, IL-7, IL-10, and IL-15 and immunophenotyping consistent with a PD-L1 ^high^, CD16 ^low^, and CD86 ^low^ cell surface expression. Treatment with LPS-EVs manufactured at both scales were effective in the ARS model, improving survival and clinical scores through improved hematopoietic recovery. EVs from unprimed MSCs were less effective than LPS-EVs, with flask EVs providing some improved survival while bioreactor EVs provide no survival benefit.

**Conclusions:**

LPS-EVs as an effective treatment for H-ARS can be produced using a scale-up development manufacturing process, representing an attractive off-the-shelf, cell-free therapy.

**Supplementary Information:**

The online version contains supplementary material available at 10.1186/s13287-024-03688-2.

## Introduction

Mesenchymal stromal cells (MSCs) represent a multi-potent population of cells with immunomodulatory properties. Despite the well-established role of MSCs in suppressing inflammatory diseases, there are only a few approved indications worldwide, including acute graft versus host disease and Crohn’s disease [1, 2]. The immunosuppressive activity of MSCs is widely thought to be mediated through their interaction with immune cells [3], which can be contact-dependent or contact-independent (e.g. secretome). MSCs secrete extracellular vesicles (EVs) which are believed to regulate monocytes/macrophages, and the direct use of EVs represents an attractive “cell-free” approach to regulating immune populations [4–7]. Advantages of therapeutic EVs compared to cell therapies include reduced immunogenicity, enhanced targeting, they are non-proliferative thus eliminating the potential of tumor formation and have the reduced manufacturing complexity in terms scalability [8]. Importantly, EVs can be stored in conventional freezers in simple storage buffers such containing protein stabilizers like trehalose [9]. We have previously shown that EVs produced from human MSCs are sufficient to educate macrophages or monocytes to be protective in mouse models of hematopoietic acute radiation syndrome (H-ARS) and musculoskeletal injuries [10–13]. Furthermore, we have observed that EVs from MSCs primed with lipopolysaccharide (LPS), a Toll-like receptor 4 (TLR-4) agonist, generate more potent macrophages and monocytes compared to EVs from unprimed MSCs [10, 11]. The direct use of EVs to treat H-ARS would be an attractive therapeutic option, but to our knowledge this has not been reported and translating these observations to the clinic has been hampered by the relative paucity of reports developing and characterizing functional EVs at a production scale.

Description of large-scale process development manufacturing of MSC-EVs have been limited [14–16] but are sorely needed to help identify critical quality control (QC) attributes [15, 17–19]. Some groups have outlined workflows for generating MSC-EVs for treatment of pancreatic cancer [20] and dendritic cell-EVs for melanoma [21]. While it is simple and cost-effective to produce EVs from MSCs on a small scale using static monolayer flask cultures for early preclinical testing, it is not conducive to large-scale production. Therefore, the development of a scalable and efficient production process is needed for the eventual advancement of clinical-grade EVs.

In this study, we investigate the functional and therapeutic properties of LPS primed MSC-EVs compared to unprimed MSC-EVs. Both EV products were produced on a large scale using a hollow-fiber bioreactor system and compared to EVs produced on a small scale using flasks by examining their physical properties and identity, gene and RNA expression, ability to educate monocytes and potency in an in vivo H-ARS mouse model. The intent of this study was not to produce EVs using identical conditions between both scales, or to produce fully characterized clinical grade EVs, but to describe a scalable developmental manufacturing process designed for clinical trials and identify key bio-potency assays that predict efficacy in the H-ARS model.

## Methods

### Isolation and flask-scale cultivation of primary MSCs

MSCs were isolated from six different human bone marrow (BM) samples (F_1, 2, 3, 4, 5, and 6) from young, healthy donors using a University of Wisconsin-Madison institutional review board (IRB)-approved protocol (2016–0298). MSCs were derived by rinsing BM filters with phosphate-buffered saline (PBS) (Hyclone, Logan, UT, USA), and isolated as described [22]. The MSCs were cultured in 75 cm^2^ plastic flasks (Greiner Bio-one, Monroe, NC) in culture media containing alpha (α) MEM media (Corning CellGro, Manassas, VA, USA) supplemented with 10% fetal bovine serum (FBS) (Hyclone, Logan, UT, USA), 100X L-Ala-L-Glutamine (GlutaGro, Corning), and 100X NEAA (Corning). MSCs used were proliferative and verified by their distinct spindle shaped morphology and adherence to plastic. MSCs were positive (approximately 95%) for MSC markers (CD29, CD44, CD73, CD90, CD105) and negative for hematopoietic markers (CD19, CD34, CD45, CD54) by flow cytometry (data not shown) as previously described [22–25].

### Flask-scale isolation of EVs and LPS-EVs from MSCs

EV isolation from MSCs at the flask-scale was performed essentially as described previously [10, 26]. Low passage number [3–6] of non-senescent frozen stocks of MSCs from the six human isolates (F_1 to F_6) were expanded in T-75 cm^2^ plastic flasks and grown to near confluence. The cells were washed with PBS (Hyclone) and replaced with MSC serum-free media (SFM) (StemPro A103332-01, ThermoFisher Scientific, Waltham, MA, USA) at 10 mL/flask. To produce LPS-EVs, MSCs from 3 isolates (F_1-F_3) were primed with LPS as described [10]. Briefly, MSCs were either unprimed (EVs) or primed with 1.0 ug/mL of *E. coli* LPS O111:B4 (L4391 Sigma, St Louis, MO, USA) (LPS-EVs) in SFM for 18–24 h. The conditioned media was collected, then centrifuged at a low-speed spin (2000 × *g* at 4 °C for 20 min) to remove any cell debris, followed by an ultra-centrifugation (UC) step (100,000 g _avg_ at 4 °C for 2 h) of the supernatant using Optima™ L-80XP Ultracentrifuge and SW28 rotor (Beckman Coulter Inc. Indianapolis, IN, USA). EVs or LPS-EVs were re-suspended in 1 ml PBS (Hyclone) per 300 mL of conditioned media, aliquoted, then stored at − 80 °C.

### Bioreactor cultivation of MSCs

MSCs were derived from Good Manufacturing Practice (GMP)-compliant human BM aspirates from a healthy donor (B_1) purchased from AllCells, (Alameda, CA, USA) and grown in MSC culture media described above but was supplemented with a more GMP-compliant xenogen-free, 5% human platelet lysate (hPL) (Millcreek, Rochester MN, USA) instead of FBS. The MSCs were characterized by morphology, flask adherence onto plastic, surface marker profile by flow cytometry (as described above) and multipotent, with the capacity to differentiate into adipogenic, osteogenic and chrondrogenic lineages in vitro, using appropriate growth factors (data not shown) [27, 28]. Large-scale cultivation of the MSC isolate (designed as B) was performed using a 200 mL Quantum hollow-fiber bioreactor (Terumo BCT, Lakewood, CA, USA). The bioreactor was initially coated with 0.005% of human fibronectin (Corning) in PBS for 4 h to aid cell adherence followed by a systemic washout with culture media. MSCs at 3.0 × 10^7^ were seeded into the bioreactor, allowed to attach for 24 h, and cells then expanded by increasing the daily media input feeding rate to compensate for the growing number of cells. The conditions to determine the optimal cell expansion in the bioreactor, including the monitoring feeding rate via glucose / lactate production, determining growth kinetics, and expansion time of the MSCs was performed as described [20, 29]. Both the sampling of the media for glucose consumption and lactate production (1.6 × 10^–8^ mmol/day) from the outer loop with calculation tables provided by manufacturer were used to monitor approximate cell numbers in the bioreactor. Peak expansion of the cells occurred after the 6-day in the bioreactor representing approx. 5 × 10^8^ cells within the bioreactor.

### Bioreactor scale isolation of EVs and LPS-EVs from MSCs

After peak expansion of MSCs in the bioreactor, expansion media was washed out with PBS and replaced with 200 mL SFM. The conditioned media was then collected at the inner loop outlet at 24-h (H) intervals (24H, 48H, 72H and 96Hup to 96 h, (24H-96H Bioreactor). The bioreactor was then extensively washed with PBS followed by a media exchange with SFM containing 1.0 ug/mL LPS O111:B4. The LPS conditioned media was then collected after 24 h (24H + LPS Bioreactor). EVs from 200 mls of conditioned media of each of these five production runs was then isolated by sequential differential centrifugation as essentially as described above using an Optima™ L-80XP Ultracentrifuge using a large capacity (> 200 mL) Ti70 rotor (Beckman Coulter Inc) at 100,000 g _avg_ at 4 °C for 2 h. EV pellets from each 24-h cycle, were resuspended, filtered through 0.22 μM filter (Terumo BCT), aliquoted and stored frozen at − 80 °C.

### EV bioreactor manufacturing scheme

**Fig. 1:**
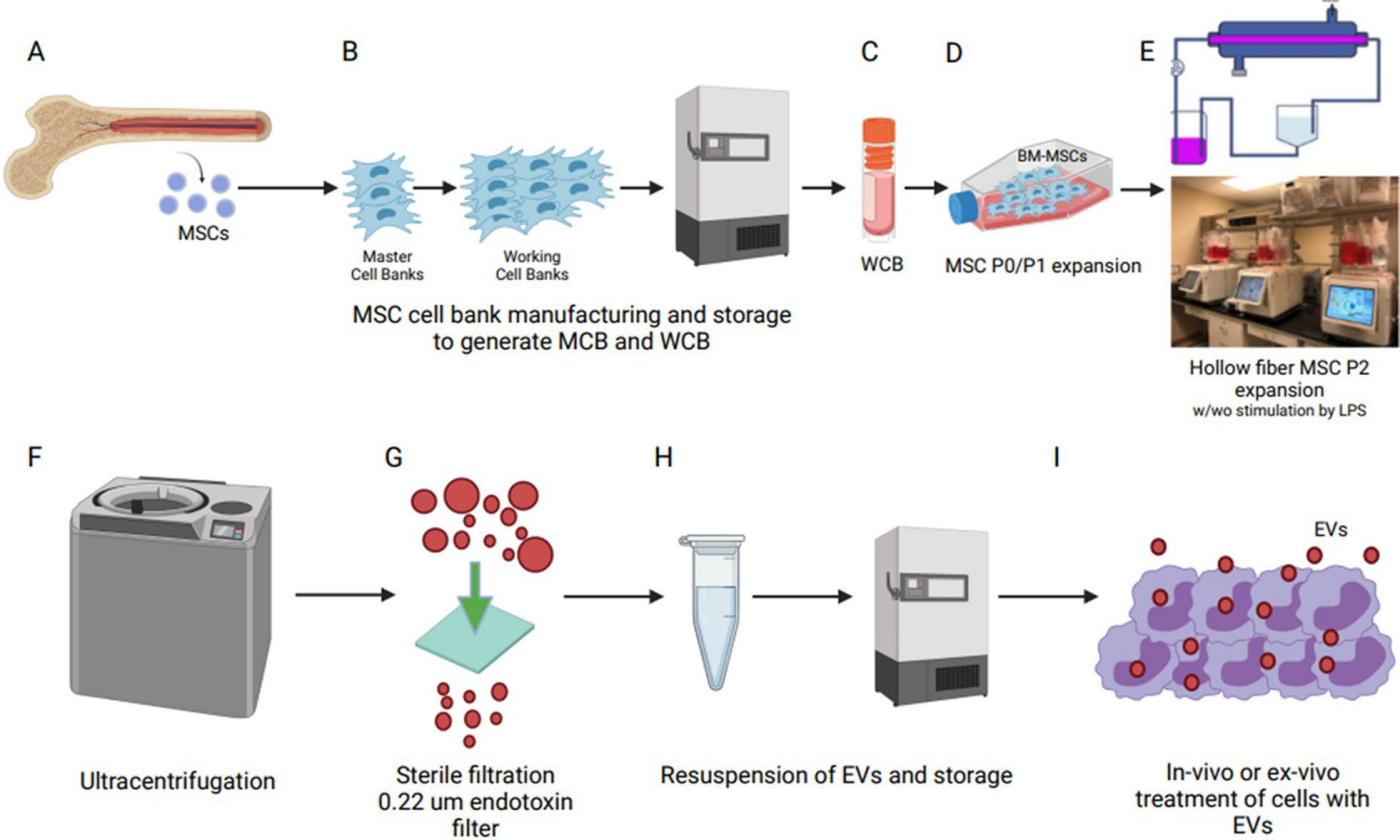
Proposed GMP manufacturing platform for MSC-EV production. **A** Mesenchymal stromal cells (MSCs) isolated from bone marrow (BM) should be characterized and qualified before **B** making a master cell bank (MCB), **C** followed by an expansion to generate multiple working cell banks (WCB). **D** Early expansion in flasks (Passage P0–P1) is followed by **E** expansion in a closed system bioreactor (P2) in serum free media. **F** EVs may be isolated directly with differential ultracentrifugation steps or concentrated beforehand using tangential flow filtration (TFF). **G** Resuspension of the EV pellet followed by sterile filtration (0.22 u) possibly with endotoxin removing capability. **H** The final EV testing and monitored storage with a consistent quality control (QC) strategy is needed to fulfill regulatory requirements for product release for **I** in vivo or ex vivo clinical testing. *Created with Biorender.com*

The use of MSC-EVs as a clinical therapeutic option is only possible if they can be reproducibly produced on a large scale. We have designed a process developmental manufacturing plan to procure therapeutic EV’s from BM MSCs (Fig. 1A–I). Human MSCs derived BM are isolated (Fig. 1A) and expanded to generate a MSC master cell bank (MCB) (Fig. 1B) and working cell bank (WCB) (Fig. 1C). For EV isolation, the WCB is expanded in flasks in suitable xenogeneic media (Fig. 1D), seeded into a bioreactor (Fig. 1E) and cell growth is monitored by determining glucose and lactate levels. The growth media exchanged with a clinical-grade SFM with a priming agent such as LPS and at 24-h intervals (24H, 48H, 72H, 96H), the conditioned medium is collected and EVs purified by differential UC [26] (Fig. 1F). The EV pellets are re-suspended, filter sterilized (0.22 um) (Fig. 1G) and assayed for physical identity (particle size range, concentration, and protein), aliquoted at an appropriate working particle concentration and stored at − 80 °C (Fig. 1H). EVs can be tested for potency by determining their ability of the EVs to educate cells ex vivo (Fig. 1I) and/or using an in vivo bioassay for efficacy in an animal model.

### RNA-seq of EVs

RNA-seq was performed on flask-scale EVs isolated from six human MSCs isolates (F_1 to F_6) and LPS-EVs from subset of 3 MSC isolates (LPS-F_1 to LPSF_3) of that group. RNA-seq was also performed on three technical replicates of the bioreactor-scale EVs (96H Bioreactor) from MSCs (isolate B). RNA-seq was performed by Systems Biosciences (Palo Alto, CA USA). Briefly, total RNA isolation was performed using the SeraMir Exosome RNA Purification Column kit (System Biosciences) and small RNA libraries were constructed with the CleanTag Small RNA Library Preparation Kit (TriLink,Biotechnologies, San Diego, CA, USA). RNA products underwent a Q/C process to ensure the cDNA products were ~ 300 nucleotides (nt) containing ~ 150nt of the RNA + 120 bp of the adaptors used to prepare libraries. The final purified library was quantified with High Sensitivity DNA Reagents and High Sensitivity DNA Chips (Agilent Technologies, Santa Clara, CA, USA). The libraries were pooled, and the 140 bp to 300 bp region was size selected on an 8% TBE gel (Invitrogen Life Technologies, Carlsbad CA, USA). The size selected library was quantified with High Sensitivity DNA 1000 Screen Tape High Sensitivity D1000 reagents (Agilent Technologies), and the TailorMix HT1 qPCR assay (SeqMatic,Fremont, CA, USA), followed by a NextSeq High Output single-end sequencing run at SR75 using NextSeq 500/550 High Output v2 kit (Illumina, San Diego, CA, USA). Small RNA concentration was performed using by Agilent Bioanalyzer Small RNA Assay using Bioanalyzer 2100 Expert instrument (Agilent Technologies).

### Characterization of the EVs and LPS-EVs

Size distribution and concentration of flask (24H Flask and 24H + LPS Flask from MSC isolates F_1 to F_6) and bioreactor particles (24H, 48H, 72H and 96H Bioreactor and 24H + LPS Bioreactor form MSC isolate B) were determined using a Nanosight NS300 instrument (Malvern Panalytical, Malvern, UK) using NTA 3.3 Dev Build 3.3.104 software and with an IZON qNano Nanoparticle Characterization instrument (IZON, Medford, MA, USA) performed by Zen-Bio Inc, (Research Triangle Park, NC, USA). Both instruments were found to give similar results. Total protein concentration as a measure of purity of EVs and LPS-EVs produced at the flask scale and bioreactor scale was also determined using NanoDrop spectrophotometer (Thermo-Fisher, Waltham, MA, USA) by Zenbio Inc, and the Bradford assay (Biorad, Hercules, CA) with BSA standards [26]. Electron microcopy on the flask EVs of several isolates performed for visual conformation has been reported [10].

### Surface marker analysis of EVs and LPS-EVs by MACSPlex flow cytometry

Surface marker profile of flask EVs and flask LPS-EVs (from two MSC isolates (F_1, 2) and the bioreactor EVs (96H-Bioreactor) and bioreactor LPS-EVs (24H + LPS Bioreactor) from MSC isolate B were determined by flow-cytometry using the MACSPlex Exosome Kit (Miltenyi Biotec, Bergisch Gladbach, Germany) which can detect 37 EV surface markers for CD105, CD11c, CD133/1, CD14, CD142, CD146, CD19, CD1c, CD2, CD20, CD209, CD24, CD25, CD29, CD3, CD31, CD326, CD4, CD40, CD41b, CD42a, CD44, CD45, CD49e, CD56, CD62P, CD63, CD69, CD8, CD81, CD86, CD9, HLA-ABC, HLA-DRDPDQ, MCSP, ROR1 and SSEA-4. This semi-quantitative assay was performed according to the manufacturer’s protocol as described [11] using a Miltenyi MACSQuant Analyzer 10 for sample acquisition and MACSQuantify Software for data analysis. The median fluorescent intensities for each surface marker were determined after subtracting fluorescent values from the respective isotype control, and values of 1.0 or more were considered positive.

### Isolation of primary human monocytes

Three human monocytes isolates were derived from peripheral blood from granulocyte colony stimulating factor (G-CSF) mobilized healthy donors as described [11] using an institutional review board (IRB)-approved protocol (2016–0298). Briefly, peripheral blood mononuclear cells (PBMCs) were first isolated using Ficoll-Paque Plus (endotoxin tested) (GE Healthcare Biosciences, Piscataway, NJ, USA) by density gradient separation. After washing with PBS (Hyclone), monocytes were isolated using anti-human CD14 microbeads (Miltenyi Biotec, Bergisch Gladbach, Germany) on an AutoMACS Pro Separator instrument (Miltenyi Biotec) as directed by the manufacturer. Cells were then aliquoted and stored in liquid nitrogen.

### Education of monocytes using EVs and LPS-EVs produced at both scales

Cryopreserved human monocytes were thawed and placed in cultivation media consisting of Iscove's modified Dulbecco's media (Gibco) supplemented with 10% human AB serum (Valley Biomedical, Winchester, VA, USA), 100 × MEM nonessential amino acids (Mediatech, Manssas VA, USA), 100 × sodium pyruvate (Mediatech),4 ug/mL human recombinant insulin (Life Technologies, Grand Island, NY, USA). For in vitro flow cytometry or qPCR studies, monocytes were plated into six-well culture plates at 1.6 × 10^6^ per well. For in vivo studies, 10^7^ cells were seeded into T-75 cm^2^ filter cap cell culture flask (Greiner Bio-One, Monroe, NC, USA). Briefly, monocytes were treated with PBS (control monocytes), EVs or LPS-EVs from flasks (F_1 isolate) or from the bioreactor (96H and 24H + LPS Bioreactor) at 5 × 10^9^ particles per 1 × 10^7^ monocytes and incubated at 37 °C with 5% CO_2_ for 18–24 h as described [11]. Monocytes educated with EVs from flasks or bioreactor were designated as flask or bioreactor EV educated monocytes (EEMos), while monocytes educated with flask or bioreactor LPS-EVs were designated as flask or bioreactor LPS-EV educated monocytes (LPS-EEMos).

### Gene expression analysis of EEMos and LPS-EEMos

Gene expression studies on synthesized cDNA (Verso, Thermo Scientific, Pittsburgh PA, USA) of RNA purified from 3 monocyte isolates educated with flasks or bioreactor EVs and LPS-EVs by SYBR Green based qPCR (Applied Biosystems, Waltham, MA, USA) as described [11]. Verified primers sets from Qiagen (Valencia, CA, USA) used were indoleamine 2,3-dioxygenase (IDO), interleukin (IL)-6, IL-8, IL-7, IL-10, IL-12, IL-15, and fibroblast growth factor-2 (FGF2). The comparative threshold cycle method (Ct) was used to calculate the mRNA levels and Ct values for the genes of interest and the glyceraldehyde 3-phosphate dehydrogenase (GAPDH) housekeeping gene (using GAPDH primer sets) were determined. Differences in the delta Ct (delta-delta Ct) of genes in EEMos and LPS EEMos were normalized to uneducated monocyte controls set at 1.0.

### Flow cytometric analysis of monocytes

The detection of cell surface markers of monocyte controls (uneducated), flask or bioreactor EEMos and LPS-EEMos were determined by flow cytometry as described [10, 11]. All antibodies were purchased from BioLegend (San Diego, CA) CD206: (15–2, cat# 321,105), CD163: (GHI/61, cat# 333,617), PD-L1: (29E.2A3, cat# 329,721), PD-L2: (24F.10C12, cat# 329,608), CD14: (HCD14, cat# 325,627), CD16: (3G8, cat# 302,025), HLA-DR: (L243, cat# 307,639), CD73: (TY/11.8, cat# 127,223), and CD86: (IT2.2, cat# 305,431). After a 20-min staining, monocytes were washed with PBS (Hyclone), then treated with Ghost Dye™ Red 780 viability dye (Tonbo Biosciences, San Diego, CA), cat# 13–0865). Stained cells were washed, assayed on an Attune™ NXT flow cytometer (Thermo Fisher Scientific) and analyzed using Flowjo™ 9.96 software (BD Biosciences, San Diego, CA).

### In vivo potency ARS model

A lethal xenogeneic ARS mouse model was performed as described [10] using NOD scid γc^−/−^ (NSG) mice (NOD.Cg-*Prkdc*^*scid*^* Il2rg*^*tm1Wjl*^/SzJ) purchased from The Jackson Laboratory, (Bar Harbor, Maine, USA). Both male and female mice between 8 and 16 weeks old were used using a protocol approved by the Animal Care and Use Committee at the University of Wisconsin-Madison (M005915). Experimental design adhered to ARRIVE guidelines (Additional file 1). On day 0, mice received a lethal dose of whole-body radiation at 4 Gray (Gy) using an X-RAD 320 X-ray irradiator (Precision X-Ray, North Branford, CT, USA) followed by a single intravenous (tail vein) treatment 4 h later with either PBS (control) or EVs or LPS-EVs from flasks (F_1 isolate) or from the bioreactor (96H and 24H + LPS Bioreactor) using 5 × 10^9^ particles in 100–200 uL PBS (Hyclone). Mice were monitored for survival, weight change and clinical scores at least 5 times a week. A clinical scoring system [30] was used based on the cumulative score of percent weight loss, posture, activity, and fur texture (scored from 0 to 2 for each criterion). Complete blood counts (CBC) in the mice were determined using a Hemavet 950FS analyzer (Drew Scientific Inc., Miami Lakes, FL) before radiation treatment (pre-rad) and on surviving mice at time periods after exposure as described [11].

### Statistical analysis

Statistics were performed using GraphPad Prism version 8.0 (GraphPad Software, San Diego, CA). Data were reported as mean ± SEM. Statistical significance for comparison particle size means, modes, yields and surface markers for EVs and LPS-EVs was determined using Welch t-test of unequal variances. Gene expression and flow-cytometry were compared using an ordinary one-way analysis of variance or Kruskal–Wallis test with the Dunn multiple-comparisons post-test. The statistical significance of clinical scores, weight changes and CBC comparison for the ARS model was determined using multiple t-test using the Holm-Sidak method. Mantel-Cox log-rank test was used for the comparison of the Kaplan–Meier survival curves. A *p* value less than 0.05 was considered statistically significant for all tests. For RNA seq analysis two gene sets comparison between flasks and bioreactor were identified as significantly different after meeting a significance threshold of FDR < 5%.

### Bioinformatics analysis of EV RNA-seq data

All the RNA-seq sequence data of EVs produced at the flask and bioreactor scale were processed through a consistent bioinformatics pipeline using the Exosome Small RNA-seq Analysis kit, (Maverix Biomics, Los Altos, CA, USA) a quality control and preprocessing software. Data quality was assessed using FASTQC [http://www.bioinformatics.babraham.ac.uk/projects/fastqc/] which is followed by read trimming and filtering using FastQMcf [http://code.google.com/p/ea-utils] and PRINSEQ [31]. Quality-filtered reads were mapped to the reference genome using Bowtie [32] and then analyzed using SAMtools [33] and Picard [http://picard.sourceforge.net]. The transformed data met Pearson correlation assumptions of linearity/ normality therefore this analysis was performed to assess the similarity of cargo (mi-RNA and protein transcripts) between EVs produced in the flasks and bioreactor. A calculated Pearson correlation coefficient ® value of 1.0 indicated 100% correlation between two EV samples. The square of R × 100 converts to R to % similarity (e.g., R = 0.9 converts to 81% similarity). Heatmaps were constructed comparing the most abundant mi-RNA and mRNA cargo of the EVs produced in the flask versus the bioreactor after applying DESeq2’s variance stabilizing transformation (VST) to the raw expression data reducing background and variability across a large range of expression values [34]. Differential expression (DE) analysis was performed using DESeq2 to identify significant differences in mi-RNA and protein transcripts of EVs produced at both scales [35]. Pathway enrichment analysis was performed using GSEA 4.1.0 [36, 37] on the size-factor normalized expression values of the 2,449 miRNA and proteins identified as differentially expressed using gene set databases obtained from MSigDB v7.2 [38, 39]. All parameters were left at their default values with the exception that gene sets instead of phenotypes were permuted due to the small number of replicates in each class [40]. RNA seq data have been deposited at the NCBI GEO under the accession number GSE255642.

## Results

### The physical properties of EVs produced at both scales were similar with significantly greater yields using the bioreactor

**Fig. 2:**
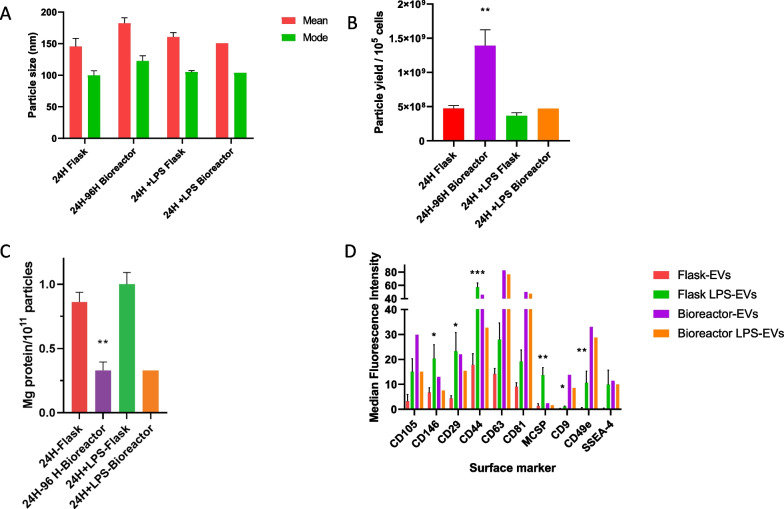
EV and LPS-EV particle size, yield, protein content and surface marker profile from flasks versus bioreactor. **A** Mean and mode (± SEM) of EV particle diameters from multiple flask production runs of EVs from conditioned media collected after 24-h (24H Flask) or after LPS stimulation (24H + LPS Flask) (*N* = 10 biological replicates) compared to EVs produced in multiple a hollow–fiber bioreactor collected after four 24-h cycles (24H-96H Bioreactor (*N* = 4 biological replicates) and after 24-h of LPS stimulation (24H + LPS Bioreactor). Overall, the mean and mode particle diameters of EVs or LPS-EVs between production methods were reproducible and not significantly different from each other. **B** Comparison of mean particle yields per 10 ^5^ cells (± SEM) from conditioned media of multiple flask runs (24H Flask and 24H + LPS Flask), (*N* = 10 biological replicates) bioreactor runs (24H-96H Bioreactor) (*N* = 4 biological replicates) and (24H + LPS Bioreactor) or with LPS stimulation and 24H + LPS Bioreactor). There was a significant (t-test) increase (*p* ≤ 0.05) in yield produced in the bioreactor runs for EVs (24H-96H Bioreactor) compared to the respective flask runs. **C** Mean protein content (± SEM) of flasks (*N* = 10 biological replicates) and bioreactor EVs (*N* = 4 biological replicates) or LPS-EVs based on mg protein / 10^11^ EV particles. The EVs of the 24H-Flask production runs had significantly more protein/ 10^11^ particles compared to the 24H-96H-Bioreactor (t-test ** *p* < 0.005). **D** Characterization of surface markers (mean (± SEM)) present on EVs (24H Flask) or LPS-EVs produced in flasks from multiple runs from MSC F1 and F2 MSC isolates (*N* = 2 biological replicates preformed in duplicate) and bioreactor MSC isolate (96H Bioreactor and 24H + LPS Bioreactor) from MSC isolate B as determined by MACSPlex flow cytometry. The EVs were stained with 37 different bead surface marker populations and compared by mean fluorescence intensity. The same set of surface markers were expressed in both EVs and LPS-EVs produced at both scales. However, when the expression levels in flask EVs and flask LPS-EVs were compared by Kruskal–Wallis with a Dunn post-test and several surface markers (CD146, CD29, CD44, MCSP, CD9 and CD49e) were found to be higher in the flask, *p ≤ 0.05, **p ≤ 0.005, ***p ≤ 0.0005

To characterize higher numbers and potentially more potent EVs from MSCs for H-ARS in a scaled-up manufacturing process, (Fig. 1) we compared EVs from small scale flask grown MSCs to large scale bioreactor grown MSCs. In addition, given prior reports by our group and others showing that TLR-4 stimulation increases EV production and potency from MSCs [11, 41], we also compared EVs isolated from LPS-primed MSCs to unprimed MSCs. As shown in Fig. 2A, the diameter (mean ± SEM and mode ± SEM) of EVs and LPS-EVs produced in flasks from multiple MSC isolates (24H Flask and 24H + LPS Flask) were reproducible and comparable at means of 146 ± 40 nm and mode 100 ± 23 nm and mean 160 ± 14 nm and mode 105 ± 5 nm, respectively. Likewise, the diameter of EVs produced in the bioreactor collected at 24H, 48H, 72H, and 96H (24H-96H Bioreactor) were all comparable with a combined mean and mode of 182 ± 17 nm and 122 ± 17 nm. The mean and mode of the bioreactor produced LPS-EVs (24H + LPS) were also similar of 151 nm and 104 nm (Fig. 2A). Particle yields (mean ± SEM) based on cell number between EVs and LPS-EVs from flasks were comparable at 4.7 × 10 ^8^ ± 3.9 × 10 ^7^ and 3.7 × 10 ^8^ ± 4.4 × 10 ^7^ particles/10 ^5^cells, respectively (Fig. 2B). However, the mean yield of the bioreactor produced EVs was threefold higher (1.4 × 10^9^ ± 2.3 × 10 ^8^ particles/10 ^5^ cells, *P* < 0.05). This yield between the flasks and bioreactor were also similar for the LPS-EVs. When yield based on conditioned media volume was also determined, the bioreactor produced far more concentrated EVs/ mL of conditioned media with up to a 38-fold increase comparing 72H and 96H Bioreactor yields (1.84 × 10 ^10^ ± 1.3 × 10 ^9^ particles/mL of conditioned media, *P* < 0.05) compared to the flask scale (24H flask) (Additional file 1: Fig. S1). This increase was also seen for the bioreactor LPS-EVs (24H + LPS Bioreactor) at 9.3 × 10 ^9^ particles/mL, or a 25.8-fold increase in yield compared to flask scale (24H + LPS Flask). The mean mg protein/ 1 × 10^11^ EV particles detected in EVs (24H-Flask) and LPS-EVs (24H + LPS Flask) from multiple flask scale runs were comparable, with mean values of 0.84 and 1.1 mg protein/ 10^11^ particles, respectively (Fig. 2C). The bioreactor produced EVs (24H-96H-Bioreactor) and LPS-EVs (24H + LPS-Bioreactor) both gave equivalent lower protein levels of 0.3 ± 0.1 mg protein/10 ^11^ particles but contained significantly less protein compared to the respective flask produced EVs. This difference is most likely due to the extra 0.22 μM filtration step performed in the bioreactor process.

When EV and LPS-EVs produced in both flasks or bioreactor were assayed for thirty-seven known surface markers by MACSPlex flow cytometry, the identities of both sets were the same, each displaying the same set of ten markers (CD105, CD146, CD29, CD44, CD63, CD81, MSCP, CD9, CD49e and SSEA-4). When comparing the expression levels of these markers between flask LPS EVs and flask EVs there was a significant increase in expression in six markers (CD146, CD29, CD44, MSCP, CD9 and CD49e) in the former (Fig. 2D). These differences were not observed between the EV or LPS-EVs produced in the bioreactor.

### RNA-seq analysis identified differences between EVs produced at each scale.

**Fig. 3:**
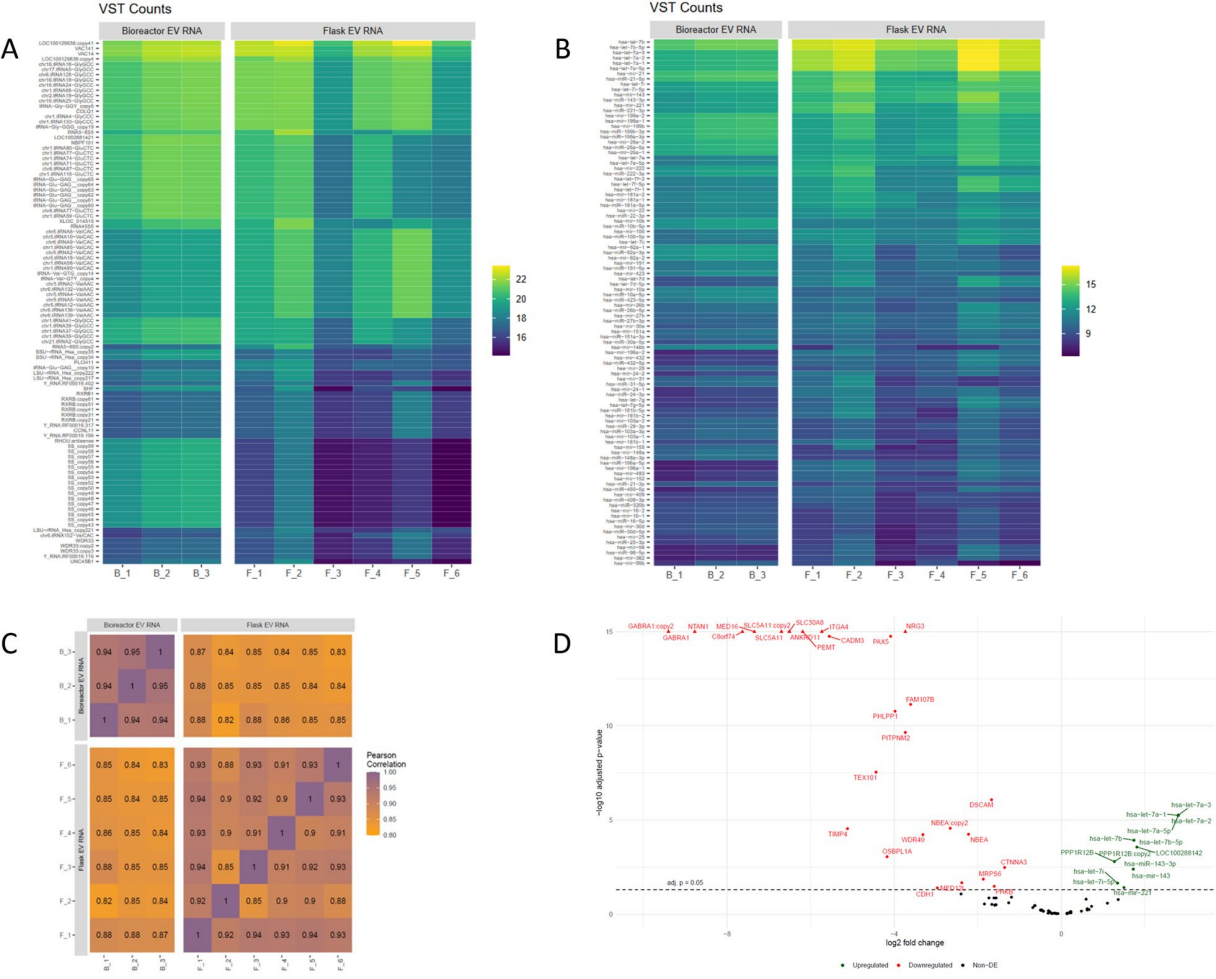
Comparison of the mi-RNA and m-RNA cargo of EVs produced by flask or bioreactor process. RNA-seq was performed on EV produced in flasks from six different MSC isolates (*N* = 6 biological replicates, F_1 to F_6) and in the bioreactor EVs (96H) of one MSC isolate **B** done in triplicate (*N* = 3, technical replicates B_1 to B_3). **A** The heatmap profile after applying variance stabilizing transformation (VST) to reduce background of the 100 most abundant messenger-RNAs and** B** micro-RNA in the flask and bioreactor EVs. **C** Correlation between the RNA-seq data sets was performed using Pearson correlation analysis, where the correlation coefficient (R) value ranges from 1.0 to 0.0, implying complete to no correlation between data sets **D** Volcano plot of significantly upregulated and down-regulated mi-RNA and m-RNA in flask EVs compared to bioreactor EVs

High-quality exosome RNA-seq libraries were successfully generated from flask EVs of six MSC isolates (F_1-F_6) and three bioreactor EVs of one MSC isolate (B_1 to B_3). RNA-seq libraries from flask LPS-EVs of three MSC isolates (LPS-F_1 to LPS-F_3) was also generated, but unfortunately the quantity of RNA transcripts from flask LPS-EVs were unacceptably low, so we focused our analysis on comparing the RNA-seq data on the EVs generated at both scales. A heat map of the 100 most abundant mRNAs transcripts (Fig. 3A) and mi-RNA (Fig. 3B) between six MSC isolates (F_1 to F_6) from flasks and the bioreactor (B_1 to B_3) indicated the RNAs between these sets were largely comparable. Since the RNA from EVs from the bioreactor (B_1 to B_3) were technical replicates, they showed stronger similarity within RNA types. The most abundant RNA transcripts within EVs generated at both small and large scales included: protein VAC14 homolog (VAC 14), Src homology 2 domain containing F (SHF), WD repeat containing protein (WDR33), and Ectodysplasin A (EDA), which all by pathway enrichment analysis mapped to intracellular signaling functions [40]. The most abundant mi-RNAs within EVs generated at both small and large scales included let-7 (a, b, i)/ miR-26 (both involved in the differentiation of cells), miR-21/ miR-143 (both tumor regulators), miR-221(angiogenesis), miR-199 (tissue formation), and protein phosphatase 1 regulatory subunit 12B (PPP1R12B) (a protein phosphorylase regulator).

Pearson correlation analysis of the RNA-seq data (Fig. 3C) to globally compare similarity of contents (mRNA transcripts, mi-RNA) within the EVs indicated that F_1 and F_3 and F_5, all showed the best correlation of 0.94 (88% similar), while MSC isolates F_2 to F_3 were less similar with a correlation score of 0.85 (72% similar). The Pearson correlation coefficients of technical replicates (B_1 to B_3) for bioreactor EVs from MSC isolate B, as expected were very similar, with correlation coefficients of 0.94–0.95 (88–90% similarity). Since this set is comprised of technical replicates of EVs from the same isolate, this coefficient value likely represents the upper limit of between two data sets. In contrast, much lower correlation values, ranging from 0.82 to 0.88 (67–77% similarity), were seen when comparing flask EVs to the bioreactor EVs, indicating larger differences in RNA content exist between EVs generated at small versus large scale.

Differential expression (DE) analysis, comparing the 100 most abundant transcripts and mi-RNAs of the grouped flask to bioreactor EVs, indicated the flask EVs showed significant increases in let-7 family miRNAs (2.5-fold more), mir-143 and mir-221 as well as PPP1R12B (Fig. 3D). A large mix of RNAs were also significantly downregulated in flask EVs compared to bioreactor EVs; many of which possessed seemingly unrelated functions, although several transcripts such as ITGA 4 (integrin alpha 4), DSCAM (DS Cell Adhesion Molecule), CTNNA3 (Catenin Alpha 3) and CDH1 (cadherin 1) encode proteins specifically involved in cell–cell adhesion. To quantitate global differences between the flask and bioreactor EVs, we found that 7715 transcripts and mi-RNA were differentially expressed, and of these, 3624 (~ 5.5%) were upregulated while 4091 (~ 6.2%) were downregulated in the flask EV groups. Out of 9247 gene sets, 2835 were upregulated in flask EVs while 6412 were upregulated in bioreactor EVs.

### LPS-EVs produced at both scales reproducibly generated alternative activated monocytes

**Fig. 4:**
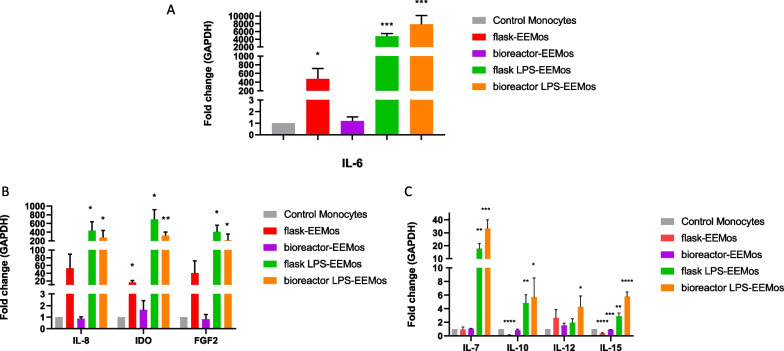
Gene expression of monocytes educated with EV or LPS-EV produced by flask or bioreactor process. Monocytes from 3 isolates were educated with flask (F_1) or bioreactor (B_1) produced EVs or LPS-EVs flask to generate flask EEMos, flask LPS-EEMos, bioreactor EEMos, or bioreactor LPS-EEMos After education, monocytes were collected, RNA isolated and analyzed by RT-PCR for gene expression (*N* = 3 to 6 biological replicates for each isolate). The fold-change of gene expression (± SEM) normalized to a GAPDH housekeeping gene and compared to untreated control monocytes **A** IL-6, **B** IL-8, IDO, FGF2 and **C** IL-7, IL-10, IL-12, and IL-15. Groups compared by Kruskal–Wallis with a Dunn’s post-test, *p < / = 0.05, ** *p* < / = 0.005, *** *p* < / = 0.0005 **** *p* < / = 0.0001

Beyond comparing EV size, surface marker profile, and RNA content, we next wanted to measure in vitro potency by assessing their ability to educate monocytes. In our experience, exosome educated monocytes (EEMos) have a unique gene expression profile and protein cell surface immunophenotype. LPS-EVs generated from both small and large scales produced LPS-EEMos with a similar gene expression profile. Compared to control monocytes, IL-6 was found to be significantly elevated (approximately 5000-fold) (Fig. 4A) with significant increases in IL-8, IDO, FGF-2, IL-7, IL-10, and IL-15 gene expression also noted (Figs. 4B–C) in EEMos generated from flask and bioreactor LPS-EVs. Except for IL-12, which was significantly higher in only LPS-EEMos generated from the bioreactor, the gene expression profile of the LPS-EEMos using either flask or bioreactor LPS-EVs were not significantly different from each other. Using flask EVs, the gene expression profile of the flask-EEMos showed some similarities to the LPS-EEMos, with lower increases in IL-6 and IDO, but was also unique with lower expression of IL-10 and IL-15. In contrast, the profile in the bioreactor EEMos using the bioreactor EVs were quite different from the other groups, with no significant change in expression except for IL-15 when compared to the control monocytes (Fig. 4A–C).

**Fig. 5:**
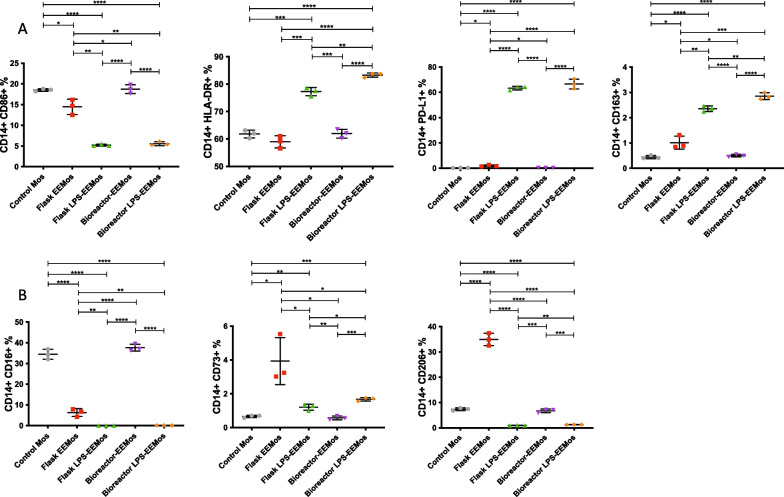
Flow cytometric analysis of human monocytes educated with EVs or LPS-EVs produced by flasks or bioreactor. Monocytes from 3 isolates were educated with flask EVs (24H flask or 24H + LPS flask) from the F_1 MSC isolate, or bioreactor produced EVs (96H Bioreactor or 24H + LPS Bioreactor) from the B MSC isolate to generate flask EEMos, bioreactor EEMos, flask LPS-EEMos or bioreactor LPS-EEMos. Cells were analyzed by flow cytometry (*N* = 3 biological replicates). The percent (%) CD14^+^ cells for each marker (± SEM) is shown **A** CD86, HLA-DR, PD-L1 and CD163 **B** CD16, CD73 and CD206. Groups were compared by Kruskal–Wallis with a Dunn post-test *p < / = 0.05, ** *p* < / = 0.005, *** *p* < / = 0.0005 **** p < / = 0.0001 between groups is designated by bars as compared to control monocytes

The proteomic cell surface marker profile of monocytes educated by EVs or LPS-EVs at both small and large scales indicated that LPS-EEMos using flask or bioreactor LPS-EVs were comparable (Fig. 5); both showed significant increases in the immunomodulating marker, PD-L1, the M2 marker, CD163, along with significant decreases in pro-inflammatory markers, CD16 and CD86 (Fig. 5A–B) compared to control monocytes. There were also some differences; LPS-EEMos generated by large scale bioreactor showed significant increases in the M1 marker HLA-DR, and M2 markers CD163 and CD206 compared to LPS-EEMos generated by small scale flasks. Interestingly, compared to all other groups, the EEMos generated by flasks uniquely expressed significantly higher M2 markers, especially CD73 and CD206 (Fig. 5B). Notably, higher levels of CD16, present in control monocytes, was also detected in EEMos generated by bioreactor, in contrast to EEMos generated flasks and LPS-EEMos generated by flasks or bioreactor.

### Treatment of lethally irradiated mice with LPS-EVs produced from either scale is effective against H-ARS

**Fig. 6:**
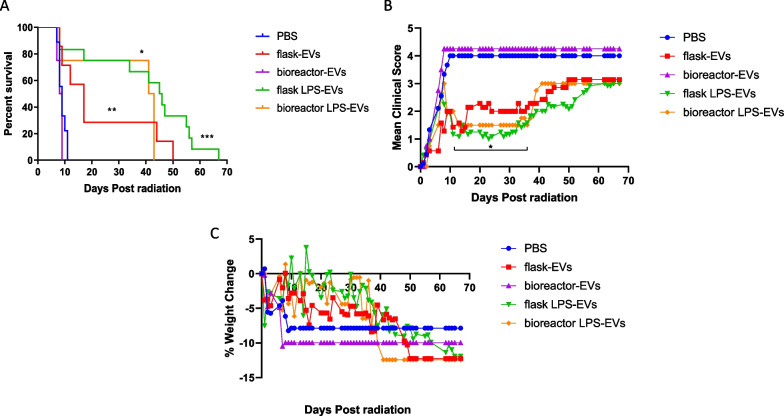
Treatment with EVs or LPS-EVs from flask or bioreactor in mice after lethal ARS. On day 0, NSG mice received 4 Gy of lethal radiation followed by an i.v. treatment 4 h later with vehicle control (PBS), or 5 × 10 ^9^ of flask-EVs, flask LPS-EVs (24H flask or 24H + LPS flask) from the F_1 MSC isolate, bioreactor-EVs, or bioreactor LPS-EVs (96H Bioreactor or 24H + LPS Bioreactor) from the B MSC isolate. Mice were followed for **A** overall survival, **B** clinical scores (percent weight loss, posture, activity, and fur texture) and **C** percent weight change. The final mean percent weight change and clinical score were carried over after death to allow for comparison by Kruskal–Wallis with a Dunn post-test between groups at a given time point. Results pooled from three separate experiments with 4 to 12 mice/group. *p < .05, ***p ≤ .005, ****p ≤ .0001

As an in vivo potency model, NSG mice were lethally irradiated to generate H-ARS and then treated with vehicle (PBS), EVs from unprimed MSCs generated by flasks or bioreactor, or EVs from LPS-primed MSCs generated by flasks or bioreactor. A single treatment with flask or bioreactor LPS-EVs effectively protected mice against H-ARS by improving overall survival (Fig. 6A), with median survival after treatment with flask or bioreactor LPS-EVs of 45.5 and 42 days, respectively, compared to 9 days for PBS treated controls. Treatment with flask or bioreactor LPS-EVs also significantly improved mean clinical scores between days 10 to 37 (*p* = 0.05 to 0.005) (Fig. 6B). Weight recovery was also notably better in these mice when compared to PBS treated controls (Figs. 6C). While greater than 75% of the mice treated with both LPS-EVs survived by day 35, the protective effect of a single infusion began to wane as both weight loss and clinical scores slowly increased between days 43 and 68 in surviving mice, suggesting potential need for repeated EV infusions. Direct treatment with EVs from unprimed MSCs generated by flasks was less potent but still promoted a significant prolongation of survival, with median survival of 17 days, with some surviving beyond day 40 (Fig. 6A) and modest improvements of clinical scores (Fig. 6B). In contrast, EVs from unprimed MSCs generated by bioreactor did not prolong survival, and like PBS treated controls, with a median survival of 8.5 days and no long-term survivors, improvements in clinical scores or weight gain. One mechanism by which treatment with LPS-EVs generated at both scales, and to a lesser extent, the EVs from unprimed MSCs generated by flask, improved survival was through acceleration of hematologic recovery as measured in peripheral blood (Additional file 1: Table S1). Recovery was not immediate, as early after irradiation (day 5–6), CBCs showed pancytopenia. But during clinical recovery (days 30–31) CBCs from mice treated with LPS-EVs generated at both scales or EVs from unprimed MSCs generated by flasks significantly improved, especially with higher white blood cells and neutrophils returning to pre-irradiation levels (Additional file 1: Table S1). Platelet counts also increased at this time, although interestingly only mice treated with EVs from unprimed MSCs generated by flasks improved to pre-irradiation levels. Since no survivors were present in mice treated with EVs from unprimed MSCs generated by bioreactor or untreated controls after day 9, CBCs could not be tracked in these groups long-term. In summary, LPS-EVs generated by both flasks or bioreactor, and to a lesser extent, EVs from unprimed MSCs generated by flasks, effectively improve hematologic and clinical recovery in mice after H-ARS.

## Discussion

In this report, we successfully establish a developmental workflow for producing large quantities of EVs from MSCs using a hollow-fiber bioreactor-based system. Importantly this system is reported to be scalable, reproducible, and GMP-compliant for other applications [42, 43]. The key advantages for EV production is that it requires relatively small volumes of media for MSC growth, enabling higher yields of EVs per mL and multiple production cycles can be more performed without subculturing compared to conventional flask formats. A primary disadvantage using a bioreactor is that it is initially a more complex system that requires a high level of skill to expand and maintain adherent cells compared to other cell-culture platforms. Since a large number of cells are grown in very low volumes, the metabolomics for optimal cell growth needs to be understood and carefully monitored to maintain the health of the cells. Plus, since a hollow fiber bioreactor relies on a porous perfusion membrane allowing the passage of nutrients and removal of waste products, any blockage can result in rapid cell death.

The large surface area of a small unit at 2.1 m^2^ (equivalent to 280 T-75 flasks) requires 14-fold less media but supports at least 25-fold more MSCs/mL in the bioreactor (2.5 × 10^6^ cells/ml) compared to flasks (10^5^ cells/mL) of equivalent surface area. This effectively concentrates EVs so more downstream EV purification options are possible, especially the volume restrictive processes like UC or size-exclusion chromatography. Useful target concentrations for production aims for a 10 to 50-fold concentration of conditioned media (personal communications). We are within range at a 38-fold concentration of EVs/mL using the bioreactor process compared to the conventional flask process. While other concentration options are possible, i.e. tangential flow filtration (TFF), they typically add costs, take more time, and through sheer forces can lead to aggregation and loss of EV integrity (unpublished results). Using UC for downstream purification, there are now GMP-compliant continuous flow UC systems capable of handling 40–100 L of media making this approach more compatible for large volumes needed for clinical trials [42, 43]. While ultimately the manufacturing configuration required for mass production depends on the clinical trial, combining a scaled-up version of the bioreactor coupled with newer UC systems may be a cost-effective approach of manufacturing functionally active EVs. Drawbacks of using UC systems include the loss of EV integrity after exposure to high G-forces, especially if multiple rounds of centrifugation are required when washing the EVs.

Differential expression analysis of the RNA-Seq data identified several mi-RNA involved in bio-potency as there was significant upregulation of several micro-RNAs found in the EVs from the flask but not the bioreactor. These included both miR-143 and the let-7 family of mi-RNAs known to post-transcriptionally silence or activate genes involved in innate immunity and can relieve the repression of several immune-modulatory cytokines such as IL-6 and IL-10 [44]. Furthermore, miR-143 mostly known to function as a tumor suppressor can also promote apoptosis of dying cells after radiation exposure [45]. Importantly, let-7 mi-RNAs can specifically down-regulate TLR-4 [46] and induce macrophages into a reparative M2-like state [47]. Intriguingly, a recent report also indicated that LPS priming of MSCs also led to increased expression of let-7b miRNA in the EVs [16]. This group also found LPS-EVs also promoted M2 macrophage activation and were effective at resolving inflammation and healing wounds. Overall, the superior potency seen in the LPS-EVs indicates that future RNA-seq studies may be informative to determine if they also possess elevated levels of these mi-RNAs.

We found that MSC priming with LPS, a TLR4 agonist, enhanced EV reproducibility between scales by eliminating measurable differences in LPS-EVs that were seen in EVs from unprimed MSCs generated at both scales. Informative QC assays for product reproducibility are essential scale-up manufacturing process of EVs. The physical identity assays (particle size and protein) used largely as defined in Minimum Information for the Study of EVs (MISEV) confirmed that EVs from unprimed or LPS-primed MSCs produced in the large-scale bioreactor were generally comparable to the respective “gold standard” set produced in small scale flasks. Of interest, several markers (CD44, CD146, CD29 and CD49) bind extracellular matrix proteins, indicating potential enhanced binding properties of LPS-EVs to damaged tissue. Also, essential to be maintained throughout scale up process, we identified several novel QC assays for EV potency. Both the gene expression profiling and cell surface marker analysis of monocytes educated ex vivo indicated that LPS-EVs generated at both scales were more comparable than EVs from unprimed MSCs. The LPS-EVs at both scales activated monocytes into a similar, radio-protective M2-like phenotype with increased expression of immuno-modulating (IL-6, IDO), anti-inflammatory (IL-10) and tissue remodeling (FGF-2, IL-7) genes. They also displayed an immunomodulating and anti-inflammatory cell surface profile (PD-L1 ^high^, CD163 ^high^, CD16 ^low^, CD86 ^low^). EEMos produced by flask EVs shared some similarities with EEMos produced by LPS-EVs, including high IL-6 expression and CD16 ^low^, but also showed distinct M2-like properties wiith CD73 ^high^, CD206 ^high^. EVs generated by bioreactor led to a more pro-inflammatory phenotype (low IL-6 and CD206 ^low^ CD16 ^high^) in EEMos. Overall, both the source of EVs (unprimed versus LPS-primed MSCs) and production scale influenced the polarization of monocytes after education. While it would have been informative to have isolated bioreactor EVs from multiple biological replicates instead of technical replicates as performed here, at this earlier stage in product development we opted to focus on the scale-up EV process by using one characterized MSC isolate. Moreover, by performing RNA-seq on bioreactor EVs from three technical replicates from a single isolate, we were also able to get a sense of the inherent variability within the RNA-seq library process. Future studies are planned to compare characterized EVs and their yields isolated from multiple MSC isolates using a more developed bioreactor/UC process.

It has been reported that priming in general can promote MSCs to a more homogenous state [48], possibly by muting any unwanted environmental stimuli. Consequently, primed MSCs respond by also producing more homogeneous EVs compared to unprimed MSCs. The utility of LPS priming may be especially important when transitioning from small to large scale manufacturing, by ameliorating any effects component changes such as changes in culture composition (e.g., FBS to hPL) may have during the process. However, given regulatory concerns of safety with potential LPS contamination in the final EV product, there is a need to explore options to reduce LPS contamination levels and/or find less toxic substitutes. The second option is especially attractive since most LPS preparations from bacteria contain low levels of contaminating *E. coli* nucleic acid and using synthetic agents would likely overcome the interference seen when preparing future RNA-seq libraries. Fortunately, there are filtration units designed to remove LPS from the final product or synthetic TLR4 priming agents to minimize unwanted side effects of LPS contamination [49].

The most important QC assay for potency involves the in vivo testing of the EVs in the H-ARS model. Efficacy of LPS-EVs generated by large scale bioreactor was similar to LPS-EVs generated by flasks, as both led to a significant enhancement of survival with improved clinical scores and weight gain in mice after lethal H-ARS. EVs from unprimed MSCs generated by flasks were partially effective in the H-ARS model and matches our recent report showing partial effectiveness of using flask-EVs to educate monocytes [11]. While protection against lethal radiation by LPS-EVs likely occurs in part from stimulating hematologic recovery of leukocytes and vital in preventing secondary infections, we believe that the *endogenous* M2-like education of macrophages/monocytes by EVs in the mouse is key for radioprotection in this model. Whether these EVs can similarly educate human monocytes in vivo, as we observed ex vivo, will have to be explored in humanized mouse models.

## Conclusions

In summary, we describe a GMP-compliant developmental bio-manufacturing process for generating EVs from both unprimed MSCs and LPS-primed MSCs and describe QC assay methods for identity and bio-potency. We found that TLR4 stimulation by LPS priming of MSCs improves both biopotency and reproducibility of EVs when produced at large scale by bioreactor and small scale by flasks. Direct treatment with LPS-EVs are an effective treatment for promoting hematopoiesis in the ARS model. While LPS-EVs is an attractive cell-free “off-the-shelf” therapy it requires further studies using safer and purer TLR-4 agonists, a better understanding of EV cargo and assessment of bio-distribution and impact on human HSCs.

### Supplementary Information

**Additional file 1. Fig. S1: **EV and LPS-EV particle yields per mL of conditioned media from flasks versus bioreactor. The mean particle yield per mL (± SEM) were generated from multiple flask production runs isolated from flasks (24H Flask) and LPS EVs (24H+LPS flask) (*N*=10 biological replicates) from three (F1_F3) MSC isolates. The bioreactor EVs (24H-96H Bioreactor) were generated from multiple production runs (*N*=4 biological replicates) or the bioreactor LPS-EVs (24H+LPS Bioreactor) after one 24-hours of LPS stimulation run from one **B** MSC isolate. There was a significant (t-test) increase (*p* ≤ 0.05) in yield per mL produced in the bioreactor runs for EVs (24H-96H Bioreactor) compared to the respective flask runs. **Table S1. **Effect of EV or LPS-EV Treatment on Complete Blood Counts after in Mice after Lethal Irradiation. Key: n/a = not applicable. * = *p* < 0.05, ** = *p* < 0.01, *** = *p* < 0.001 as compared to pre-radiation (pre-rad control). Mean CBCs ( +/- SEM) after single i.v. treatment of vehicle (PBS), of EVs made in flasks (Flask-EVs) or bioreactor (Bioreactor-EVs) and EVs from LPS-primed MSCs made in flasks (Flask LPS-EVs) or bioreactor (Bioreactor LPS-EVs).

## Data Availability

RNA seq data have been deposited at the NCBI GEO under the accession number GSE255642. The other datasets used and/or analyzed during the current study are available from the corresponding author on reasonable request.

## Abbreviations

ARS: Acute radiation syndrome
BM: Bone marrow
CBCs: Complete blood counts
CDH1: Cadherin 1
Ct: Comparative threshold
CTNNA3: Catenin alpha 3
DE: Differential expression
DSCAM: DS cell adhesion molecule
EDA: Ectodysplasin A
EEMos: Unprimed MSC-exosome educated monocytes
EVs: Extracellular vesicles
FBS: Fetal bovine serum
FGF2: Fibroblast growth factor-2
GMP: Good manufacturing practice
G-CSF: Granulocyte colony stimulating factor
GDPDH: Glyceraldehyde 3-phosphate dehydrogenase
Gy: Gray
hPL: Human platelet lysate
HSC: Hematopoietic stem cell
IDO: Indoleamine 2, 3-dioxygenase
IL: Interleukin
IRB: Institutional review board
ITGA 4: Integrin alpha 4
LPS: Lipopolysaccharide
LPS-EEMos: Lipopolysaccharide-primed
LPS-EVs: Lipopolysaccharide extracellular vesicles
MCB: Master cell bank
MEMs: MSC-educated macrophages
MSCs: Mesenchymal stromal cells
miR: MicroRNAs
NSG: NOD-SCIDγc^−/−^
PBMCs: Peripheral blood mononuclear cells
PBS: Phosphate-buffered saline
PCR: Polymerase chain reaction
PD-L1: Programmed death-ligand 1
PPP1R12B: Protein phosphatase 1 regulatory subunit 12B
QC: Quality control
qPCR: Quantitative polymerase chain reaction
RBC: Red blood cell
SFM: Serum-free media
SHF: Src homology 2 domain containing F
RNA-seq: RNA sequencing
TFF: Tangential flow filtration
TLR-4: Toll-like receptor 4
UC: Ultracentrifugation
VAC 14: Protein VAC14 homolog
WBC: White blood cell
WDR33: WD repeat containing protein
WCB: Working cell bank
