# Supplementary material for: Large-scale bioreactor production of extracellular vesicles from mesenchymal stromal cells for treatment of acute radiation syndrome
Source: Stem Cell Res Ther. 2024 Mar 13;15:72. doi: 10.1186/s13287-024-03688-2 (PMC10936083; doi:10.1186/s13287-024-03688-2)
Supplement: Supplementary file 1 — Additional file 1. Fig. S1: EV and LPS-EV particle yields per mL of conditioned media from flasks versus bioreactor. The mean particle yield per mL (± SEM) were generated from multiple flask production runs isolated from flasks (24H Flask) and LPS EVs (24H+LPS flask) (N=10 biological replicates) from three (F1_F3) MSC isolates. The bioreactor EVs (24H-96H Bioreactor) were generated from multiple production runs (N=4 biological replicates) or the bioreactor LPS-EVs (24H+LPS Bioreactor) after one 24-hours of LPS stimulation run from one B MSC isolate. There was a significant (t-test) increase (p ≤ 0.05) in yield per mL produced in the bioreactor runs for EVs (24H-96H Bioreactor) compared to the respective flask runs. Table S1. Effect of EV or LPS-EV Treatment on Complete Blood Counts after in Mice after Lethal Irradiation. Key: n/a = not applicable. * = p < 0.05, ** = p < 0.01, *** = p < 0.001 as compared to pre-radiation (pre-rad control). Mean CBCs ( +/- SEM) after single i.v. treatment of vehicle (PBS), of EVs made in flasks (Flask-EVs) or bioreactor (Bioreactor-EVs) and EVs from LPS-primed MSCs made in flasks (Flask LPS-EVs) or bioreactor (Bioreactor LPS-EVs). [file 13287_2024_3688_MOESM1_ESM.pdf]

Supplemental Figure 1

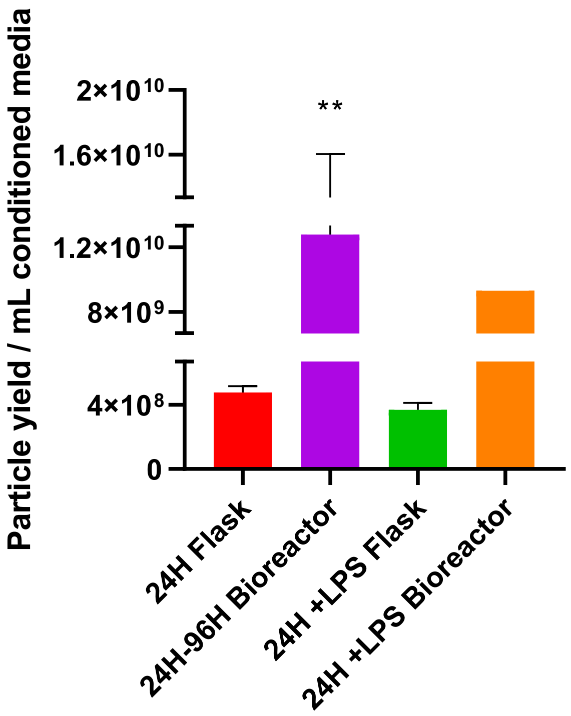

Supplemental Table 1

Group	*Day post radiation*	*RBC (M/ul)*	*WBC (K/ul)*	*Neutrophils (K/ul)*	*Lymphocytes (K/ul)*	*Monocytes (K/ul)*	*Platelets (K/ul)*	*Platelet volume (fL)*
Pre-rad Control	n/a	8.86 (0.18)	2.75 (0.33)	1.56 (0.13)	0.93 (0.19)	0.13 (0.02)	955.4 (48.1)	4.90 (0.03)
PBS	5-6	7.44 (0.57)*	0.36 (0.08)**	0.09 (0.03)***	0.17 (0.06)*	0.02 (0.01)*	164.6 (32.6)***	5.61 (0.33)**
Flask-EVs	5-6	8.67 (1.0)	0.27 (0.03)*	0.04 (0.01)**	0.14 (0.04)	0.02 (0.01)	175.0 (31.5)***	5.50 (0.53)*
Bioreactor-EVs	5-6	9.67 (0.81)	0.25 (0.04)*	0.04 (0.02)**	0.07 (0.06)	0.01 (0.003)	175.3 (34.9)***	5.73 (0.54)**
Flask LPS - EVs	5-6	8.82 (0.58)	0.25 (0.05)***	0.05 (0.01)***	0.09 (0.02)*	0.02** (0.002)	134.2 (14.6)***	6.65 (0.13)***
Bioreactor LPS-EVs	5-6	9.72 (1.0)	0.26 (0.05)*	0.05 (0.02)**	0.11 (0.05)	0.03 (0.02)	180.0 (33.0)***	6.50 (0.17)**
Flask-EVs	30-31	7.47 (0.06)	2.3 (0.13)	0.84 (0.07)	1.26 (0.04)	0.2 (0.04)	876.7 (113.0)	5.75 (0.07)***
Flask LPS - EVs	30-31	7.78 (0.29)	1.87 (0.42)	1.47 (0.42)	0.30 (0.06)	0.07 (0.02)	487.6 (80.2)***	5.76 (0.15)***
Bioreactor LPS-EVs	30-31	8.64 (0.47)	1.59 (0.11)	1.36 (0.08)	0.19 (0.04)	0.17 (0.003)	438.7 (115.4)*	5.77 (0.32)***
